# Analysis of cellular NO-GC expression in the murine heart and lineage determination in angiotensin II-induced fibrosis

**DOI:** 10.1016/j.isci.2024.111615

**Published:** 2024-12-16

**Authors:** Lennart Kreutz, Annika Gaab, Malathi Dona, Alexander R. Pinto, Michelle D. Tallquist, Dieter Groneberg, Andreas Friebe

**Affiliations:** 1 Physiologisches Institut, Julius-Maximilians-Universität Würzburg, Würzburg, Germany; 2 Baker Heart and Diabetes Institute, Melbourne, VIC, Australia; 3 Center for Cardiovascular Research, University of Hawaii at Manoa, Honolulu, HI, USA; 4 Translational Center for Regenerative Therapies (TLC-RT), Fraunhofer Institute for Silicate Research (ISC), 97082 Würzburg, Germany

**Keywords:** Rodent cardiology, Biological sciences, Cell biology

## Abstract

NO-sensitive guanylyl cyclase (NO-GC) is involved in the (patho)physiology of the mammalian heart. However, little is known about the individual cardiac cell types that express NO-GC and the role of the enzyme in cardiac fibrosis. Here, we describe the cellular expression of NO-GC in healthy and fibrotic murine myocardium; these data were compared with scRNA-seq data. In healthy myocardium, NO-GC is strongly expressed in pericytes and smooth muscle cells but not in endothelial cells or cardiomyocytes. Angiotensin II induced cardiac hypertrophy and fibrosis; fibrotic lesions contained cells positive for NO-GC identified as activated fibroblasts. Lineage tracing indicates that NO-GC-expressing activated fibroblasts originate from PDGFRβ- and Tcf21-positive fibroblast precursors. Our data indicate NO-GC expression in cardiac pericytes and SMC in naive myocardium and in activated fibroblast in fibrotic heart tissue. NO-mediated signaling may modulate fibrotic responses underlying the action of NO-GC stimulators used in the therapy of heart failure.

## Highlights

• NO-GC is high in cardiac pericytes and SMC but very low in fibroblasts
• Induction of cardiac fibrosis leads to accumulation of NO-GC^+^ activated fibroblasts
• NO-GC-expressing cells in fibrosis derive from fibroblasts, not from pericytes or SMC

## Graphical abstract

**Figure:**
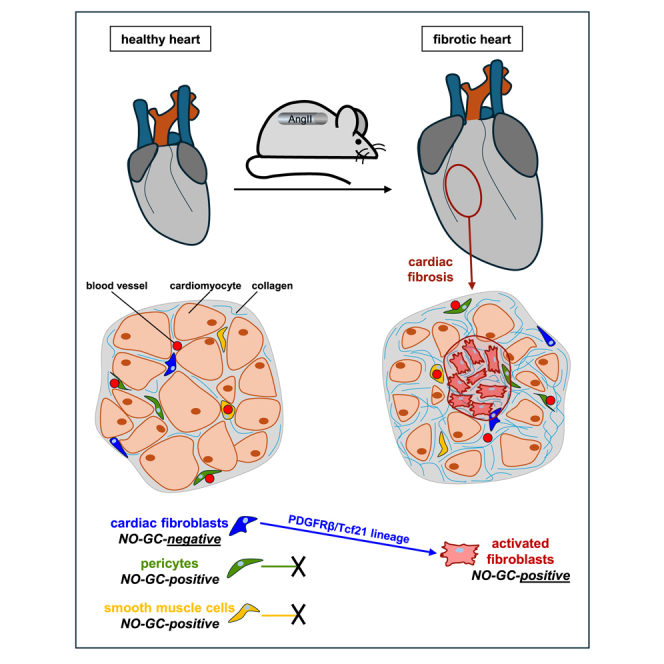

## Introduction

Nitric oxide-sensitive guanylyl cyclase (NO-GC) is a heterodimeric enzyme consisting of an α and a β subunit. There are two isoenzymes, NO-GC1 and NO-GC2 (α_1_β_1_ and α_2_β_1_), respectively.^1^ NO-GC1 is the predominant form in the cardiovascular system whereas NO-GC2 is primarily expressed in the neuronal system.^2^^,^^3^ Nitric oxide (NO) is the physiological activator of the enzyme. By catalyzing the conversion of guanosine triphosphate to cyclic guanosine monophosphate (cGMP), NO-GC mediates numerous physiological and pathophysiological processes. Stimulation of cGMP production (e.g., with the NO-GC stimulator vericiguat) is in fact used as pharmacological approach to treat patients with heart failure with reduced ejection fraction.^4^^,^^5^^,^^6^ The exact cell type(s) in which vericiguat exerts its beneficial effects are not known.

Cardiac tissue composition is specialized to integrate contractile and electrical functions in order to guarantee regular blood flow throughout the body. The most frequent cells in the human ventricle are cardiomyocytes, mural cells (SMC and pericytes), fibroblasts, endothelial cells, and immune cells, yet the relative numbers are still being discussed.^7^^,^^8^ These cardiac cells are embedded in a network of extracellular matrix (ECM), predominantly made up of fibrillar collagen which is synthesized by fibroblasts.^9^ A comprehensive description of cellular NO-GC expression in the heart has not been published to date, although SMC and pericytes (similar to other organs) were shown to express the NO receptor.^10^ Thus, cell-specific effects of NO-GC are not yet understood.

Cardiac fibrosis plays a major role in heart failure.^11^ Fibrotic remodeling involves the excess deposition of collagen and other ECM proteins.^12^ Activated fibroblasts/myofibroblasts are frequently highlighted as key contributors to cardiac fibrosis. These cells produce collagen and other matricellular proteins and interact with inflammatory cells.^13^ Myofibroblasts are primarily identified by the marker α smooth muscle actin (αSMA), which signifies their contractile capability.^14^ While αSMA-positive myofibroblasts are observed in the early stages following myocardial infarction, they are absent at later time points in other models such as transverse aortic constriction (TAC), e.g., at day 14 or 28,^15^ and angiotensin II (AngII) infusion at day 14.^16^ Instead, these models exhibit clusters of αSMA-negative activated fibroblasts that actively produce ECM components, such as collagen and thrombospondin 4 (THBS4).^17^ The ECM protein THBS4, identified through scRNA-seq analyses as a specific marker for activated fibroblasts,^16^ plays a critical role in cardiac fibrosis, as evidenced by studies on THBS4-knockout mice.^18^^,^^19^

Here, we compare cellular NO-GC expression from immunofluorescence with scRNA-seq data in healthy murine heart and after induction of cardiac fibrosis by AngII treatment. As we identified NO-GC-expressing activated fibroblasts in the fibrotic scar, we used 4 different Cre lines for lineage tracing to identify the progenitors of these cells. Our results rule out SMC and pericytes as precursors of these activated NO-GC-positive cells. Rather, these activated fibroblasts appear to originate from a Tcf21/PDGFRβ-positive lineage.

## Results

### Identification of an NO-GC-expressing cell types in the murine heart

**Figure 1:**
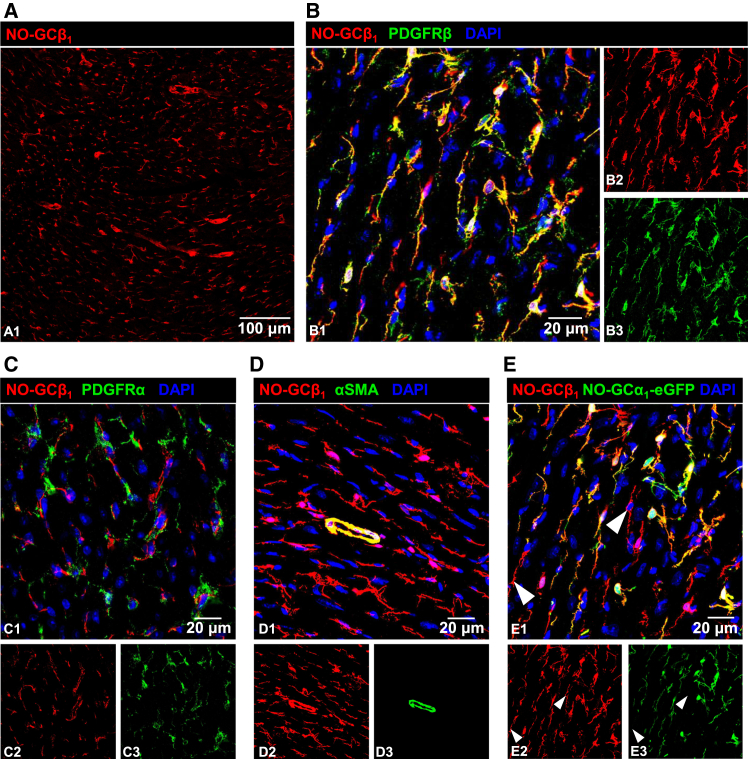
Cellular expression of NO-GC in healthy murine myocardium Cardiac tissue from healthy adult mice was obtained, fixed and immunofluorescence was performed with the indicated antibodies. (A) NO-GCβ_1_ (red) indicates β_1_ subunit expression. (B) PDGFRβ (green) co-localizes with NO-GCβ_1_, whereas PDGFRα (C, green) does not. (D) Co-localization with αSMA indicates NO-GCβ_1_ expression in vascular SMC. (E) Co-localization of the β_1_ antibody signal with NO-GCα_1_-eGFP can be seen in most cardiac cells; however, few β_1_-positive cells do not stain for NO-GCα_1_-eGFP suggesting presence of the α_2_β_1_ dimer (arrow heads). Note that (B) and (E) show identical tissue which was probed with the two indicated antibodies and expressed eGFP under control of the NO-GCα_1_ promoter). DAPI indicates cell nuclei (blue). Single channels are shown in b2/3, c2/3, d2/3 and e2/3.

The exact cellular expression pattern of the NO receptor NO-sensitive guanylyl cyclase (NO-GC) in the heart is still not entirely elucidated. Here, we used a combination of specific antibodies in immunofluorescence and compared these data with scRNA-seq data (^16^; https://www.ebi.ac.uk/gxa/sc/experiments/E-MTAB-8810/results/tsne). NO-GC was identified using an antibody against the β_1_ subunit validated against cardiac tissue from global NO-GC knockout (GCKO) mice. The absence of antibody signal in the knockout tissue confirms the antibody’s specificity (Figure S1) allowing the identification of both existing NO-GC isoforms (NO-GC1 and NO-GC2; α_1_β_1_ and α_2_β_1_, respectively) in WT heart. Figure 1A shows cardiac NO-GC expression in longitudinal tube-like structures and in small cells with long, thin extensions. All NO-GC-positive cells co-expressed PDGFRβ (Figure 1B), a marker of mesenchymal cells; thus, NO-GC/PDGFRβ are co-expressed in vascular SMC (VSMC) of the cardiac vasculature and in pericytes as indicated by their small asterisk-like shape. NO-GC in fibroblasts is scarce as shown by non-congruent signals with PDGFRα, a specific fibroblast marker (Figure 1C). As expected, alpha-smooth muscle actin (αSMA), a well-established marker for SMC, co-stained vascular structures with NO-GC indicating smooth muscle expression but not in surrounding pericytes (Figure 1D). Using a transgenic mouse line that expresses enhanced green fluorescent protein (eGFP) under the control of the NO-GCα_1_ promoter (NO-GCα_1_-eGFP; B6; FVB-Tg(Gucy1a3-EGFP)HM112Gsat/Mmucd), we can show that most cells express the α_1_β_1_ heterodimer; only in very few cells we observed NO-GCβ_1_ expression without the α_1_ subunit indicating presence of the α_2_β_1_ isoform (Figure 1E).

**Figure 2:**
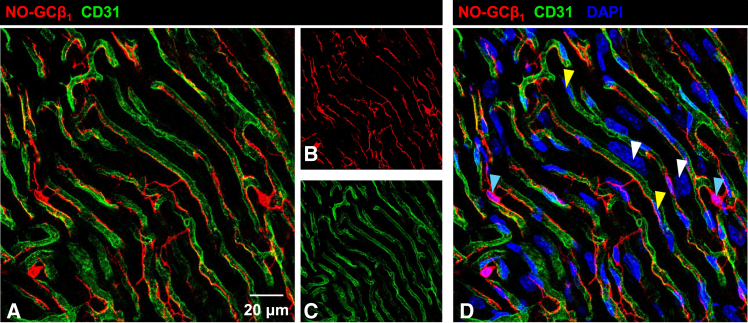
Expression of NO-GC in cardiac pericytes Cardiac tissue from healthy adult mice was obtained, fixed and immunofluorescence was performed with the indicated antibodies. (A) NO-GCβ_1_ (red) indicates β_1_ subunit expression, CD31 (green) stains endothelium. Single channels are shown in (B and C); (D) includes DAPI staining to identify cell nuclei (blue) to better distinguish cardiomyocytes (white arrowheads). Yellow arrowheads indicate nuclei from endothelial cells, light blue arrowheads indicate nuclei from pericytes.

To clarify pericytes as most abundant NO-GC-expressing cardiac cell type, we co-stained with an antibody against CD31 (cluster of differentiation 31), also known as platelet endothelial cell adhesion molecule 1, an established endothelial cell marker.^20^ CD31 clearly visualizes the capillary tubes with NO-GC-positive cells wrapped around them (Figure 2). Shape and 3D structure of these cells make them highly likely to be pericytes. In between, the cardiomyocytes, identifiable by their large nuclei, do not exhibit NO-GC expression as judged by immunohistochemistry (Figure 2 right panel).

To validate our findings, we then assessed NO-GC expression in murine myocardium using scRNA-seq.^16^ In healthy myocardium, pericytes and VSMC exhibited strong GUCY1B1 mRNA expression, the gene encoding the β_1_ subunit of NO-GC (Figure S2). Minimal expression was observed in the endocardium and resident fibroblasts. A very similar distribution was seen for the α_1_ subunit (GUCY1A1) whereas the α_2_ subunit (GUCY1A2) was barely detected. These data match our immunofluorescence data (see Figure 1E) and indicate the α_1_β_1_ isoform (NO-GC1) to be the most prominent isoform in cardiac cells.

### NO-GC expression under fibrotic conditions induced by AngII

**Figure 3:**
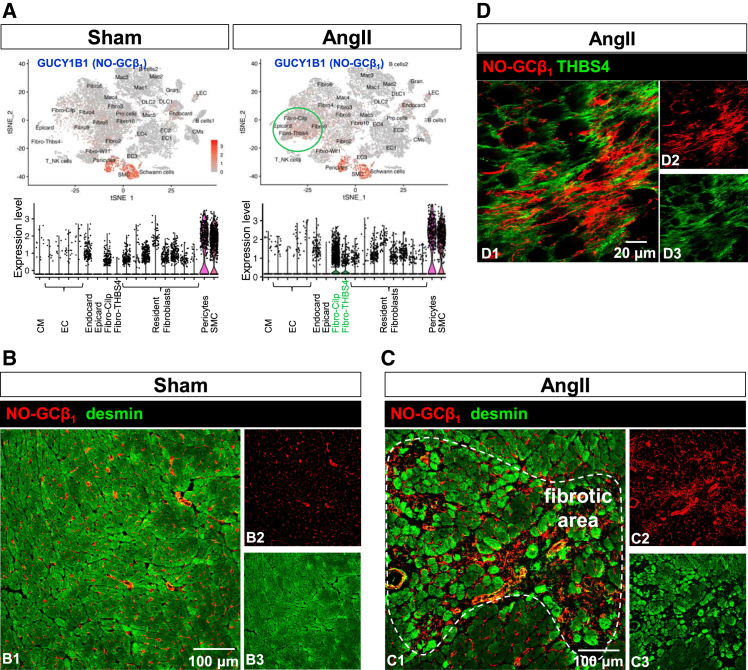
NO-GC is expressed in fibrotic areas after AngII-treatment Expression of NO-GCβ_1_ (red) and desmin (green, marking cardiomyocytes) in murine hearts. (A) scRNA-seq data for GUCY1B1 (gene for NO-GCβ_1_). (B) intact cardiac tissue from sham-treated mice show NO-GC-positive cells between cardiomyocytes. (C) disrupted myocardial architecture after AngII treatment. Surrounding the fibrotic area (indicated by dotted line), an increased expression of desmin in cardiomyocytes was observed. (D) NO-GCβ_1_-expressing cells are surrounded by THBS4. Single channels are shown in a2/3, a5/6 and d2/3. scRNA-seq data from McLellan et al., 2020; https://www.ebi.ac.uk/gxa/sc/experiments/E-MTAB-8810/results/cell-plots.

Comparison of scRNA from healthy vs. AngII-treated cardiac tissue has shown the contribution of fibroblast subtypes to the pathologic remodeling. One type of fibroblast which was positive for thrombospondin-4 (THBS4;^15^^,^^16^) was not present in untreated hearts but emerged after AngII treatment to promote fibrosis (Figure 3A). Interestingly, this newly formed fibroblast subtype is positive for NO-GC (Figure 3A). To identify this novel type of NO-GC-expressing activated fibroblast in murine myocardium, we induced cardiac fibrosis by the application of AngII via osmotic minipumps (2 mg/kg/d for 14 days). Desmin, an intermediate filament of muscle cells,^21^ was used to stain cardiomyocytes in order to visualize the disruption of cardiac architecture. Compared to sham treated controls (Figure 3B), hearts from AngII-treated mice were found to exhibit significant structural alterations, characterized by the formation of inhomogeneities in cardiomyocyte arrangement and disruption of myocardial integrity (Figure 3C). Treatment with AngII led to an increase in desmin expression,^22^ particularly noted in cardiomyocytes near fibrotic areas (Figure 3C1, dotted white line). In fact, we were able to identify increased occupancy with NO-GC-expressing cells in fibrotic regions compared to healthy heart (compare Figure 3C2 to B2). Further immunofluorescence analysis showed that THBS4, an ECM protein, was strongly expressed in fibrotic areas and that NO-GC-positive cells were surrounded by THBS4. This observation, together with the identification of Fibro-THBS4 cells as NO-GC-positive in scRNA-seq, suggests that these cells may produce THBS4 (Figure 3D; scRNA seq data in Figure S3). To validate its specificity for fibrosis, THBS4 expression was also monitored in healthy murine myocardium (Figure S4A) and found only in the aortic valve.^23^ In contrast, strong THBS4 signaling was observed in the myocardium after AngII treatment, with more intense signals in the inner layers (Figure S4B). Taken together, cardiac NO-GC expression increased in fibrotic areas after AngII treatment; proximity to THBS4, an ECM protein generated by the newly acquired NO-GC-expressing fibroblast subtypes (THBS4 fibroblast and Cilp fibroblast, both absent in naive heart) suggests that these cells migrate into and/or proliferate within the fibrotic scar.

### Evaluation of different Cre lines for lineage tracing of NO-GC expressing cells

**Figure 4:**
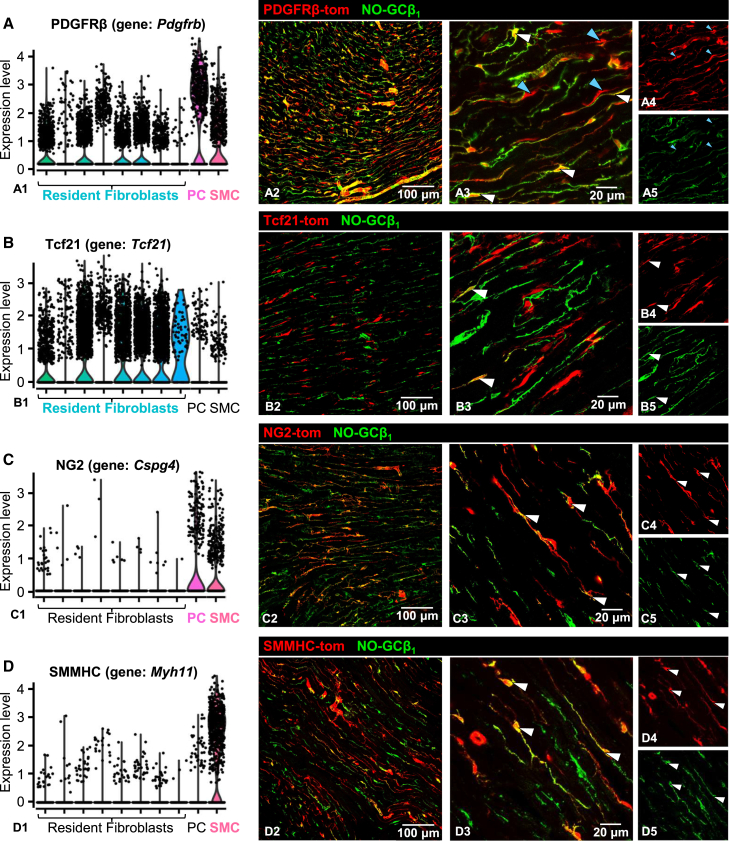
Analysis of tdTomato and NO-GC expression in four reporter mice (A–D) scRNA-seq data and respective Cre-mediated tdTomato expression for PDGFRβ (gene: *Pdgfrb*, (A), Tcf21 (gene: *Tcf21*, (B), NG2 (gene: *Cspg4*, (C), and SMMHC (gene: *Myh11*, (D), in healthy murine hearts. tdTomato (red) and NO-GCβ_1_ (green) show co-localization depending on the Cre line used. White arrowheads indicate co-localization, blue arrowheads show non-co-localized proteins. Single channels are shown in a4/5, b4/5, c4/5 and d4/5. scRNA-seq data from McLellan et al., 2020; https://www.ebi.ac.uk/gxa/sc/experiments/E-MTAB-8810/results/cell-plots.

**Figure 5:**
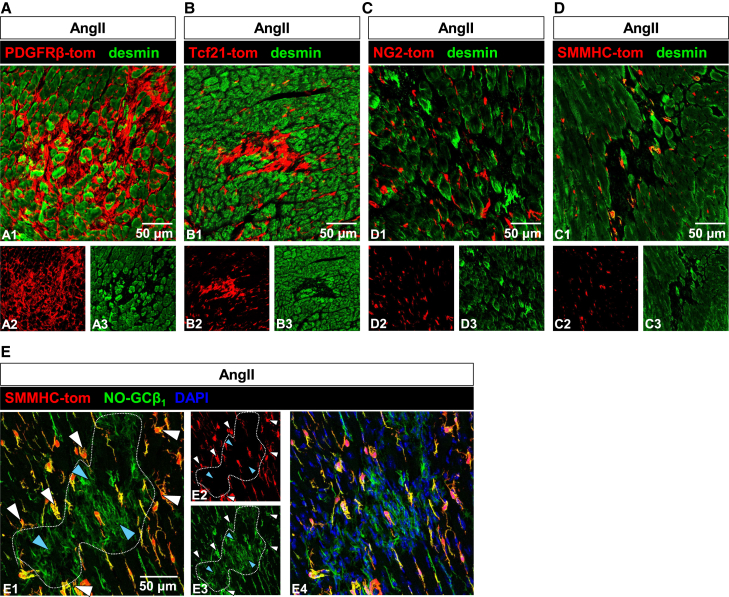
Cre-mediated expression of the tdTomato reporter after induction of cardiac fibrosis (A–E) Expression of Cre line-dependent tdTomato (red) and desmin (green, cardiomyocytes) in AngII-treated murine hearts. Expression of tdTomato was tamoxifen-induced under control of PDGFRβ-CreER^T2^ (A), Tcf21^mCrem^ (B), NG2-CreER^T2^ (C), and SMMHC-CreER^T2^ (D). (E) SMMHC-tdTomato (red) and NO-GCβ_1_ (green) show co-localization in pericytes (white arrowheads) but NO-GCβ_1_-positive, tdTomato-negative cells (blue arrowheads) are found within the fibrotic area (dotted line). DAPI (blue) is included in e4 to show accumulation of cell nuclei in the fibrotic area. Single channels are shown in a2/3, b2/3, c2/3, d2/3 and e2/3.

Following the identification of putative NO-GC-positive activated fibroblasts in fibrotic regions, we set out to uncover their origin. Two main hypotheses were proposed: first, that NO-GC-expressing cells, such as pericytes and VSMCs, migrate into fibrotic areas and differentiate into activated fibroblasts; and second, that other precursor cells, like resident fibroblasts, become activated and express NO-GC *de novo*. To investigate these possibilities, lineage tracing was performed using a tdTomato reporter strain in conjunction with four different inducible CreER^T2^ transgenic mouse lines, namely PDGFRβ-, Tcf21-, NG2-, and SMMHC-CreER^T2^. These Cre lines were chosen based on scRNA analysis (Figure 4) which indicated them to be active in SMC, pericytes and resident fibroblasts (PDGFRβ-CreER^T2^; Figure 4A1), only in resident fibroblasts (Tcf21^mCrem^; Figure 4B1), in subsets of pericytes and SMC (NG2-CreER^T2^; Figure 4C1), and in SMC and pericytes (SMMHC-CreER^T2^; Figure 4D1). It should be noted that, despite the low mRNA seen in scRNA data (see Figure 4D1), several labs including ours have shown that the SMMHC-CreER^T2^ also targets pericytes (see also Figure 5E below). Lineage tracing of tdTomato reporter mice was performed according to the scheme shown in Figure S5. In specific, mice aged 6 weeks were injected with tamoxifen on 5 consecutive days to induce the expression of tdTomato under control of the respective promoter. After 45 days, minipumps containing saline or AngII (2 mg/kg/d) were implanted. 14 days later, mice were sacrificed, and hearts were collected.

We first analyzed tdTomato expression and possible co-localization with NO-GC in the cardiac tissue under healthy conditions (Figure 4). In the PDGFRβ/tdTomato reporter mouse, all cells labeled by the NO-GCβ_1_ antibody were tdTomato-positive (Figure 4A2–A5). Very few tdTomato-positive were observed that showed no NO-GC expression (blue arrowheads in Figure 4A3–A5). In addition, complete co-localization between PDGFRβ immunosignals and PDGFRβ-tdTomato expression was also demonstrated (Figure S6A). This indicates that PDGFRβ-induced tdTomato staining mostly represents NO-GC expression. PDGFRα, as marker for fibroblasts, was also found to colocalize with PDGFRβ-tdTomato, albeit in few cells (Figure S6B, white arrowheads).

In contrast, co-localization of Tcf21-tdTomato and NO-GCβ_1_ was generally not observed (Figure 4B2). Rarely did we see double-positive cells (Figure 4B3–B5). In addition, Tcf21-tdTomato was found to be co-expressed with PDGFRβ only in some cells whereas it was identified in most of the PDGFRα-positive cells (Figures S6C and S6D). The scRNA-seq analysis of Tcf21 confirmed labeling of resident fibroblasts, with minimal expression in pericytes and VSMC (Figure 4B1). Thus, these rare double-labeled cells might either be resident fibroblasts with minor NO-GC content or a minority of pericytes with Tcf21 expression. The NG2 (gene: CSPG4) reporter mouse revealed that some but not all cells co-localized NG2-tdTomato with NO-GC (Figure 4C2) or PDGFRβ (Figure S6E); however, co-localization was not observed between NG2-tdTomato and PDGFRα (Figure S6F). The scRNA-seq analysis confirmed that this reporter mouse labeled pericytes and VSMC, but not resident fibroblasts (see Figure 4C1). Finally, in the SMMHC-tdTomato reporter, co-localization of tdTomato with NO-GC was observed (Figure 4D), and, expectedly, with PDGFRβ whereas co-localization of tdTomato and PDGFRα was not detected (Figures S6G and S6H). Taken together, using the PDGFRβ reporter, most frequent tdTomato labeling of NO-GC-expressing cells was detected whereas least co-localization was observed with the Tcf21 reporter.

### Origin of NO-GC-positive cells in fibrotic foci

Subsequently, the origin of tdTomato-positive cells in fibrotic areas was investigated. After two weeks of AngII treatment, cardiac tissue was stained with an antibody against desmin to identify cardiomyocytes thereby visualizing fibrotic scars (Figure 5). Fibrosis-associated tdTomato was found only in PDGFRβ- and Tcf21-reporter mice (Figures 5A and 5B), but not in NG2-and SMMHC-tdTomato mice (Figures 5C and 5D). Thus, SMC and pericytes are unlikely precursor cells for the NO-GC-expressing cells in the fibrotic scar. Rather, resident fibroblasts positive for PDGFRβ and Tcf21 are likely contributors to the pool of activated fibroblasts induced by AngII treatment. Analysis of fibrotic areas in SMMHC-tdTomato mice showed few NO-GC/tdTomato-positive cells (Figure 5E, white arrowheads; presumably pericytes based on their shape and scattered distribution) and densely accumulated NO-GC-positive but tdTomato-negative cells in the fibrotic scar (Figure 5E, blue arrowheads). Compared to the pericytes, these fibrosis-associated cells showed a more diffuse NO-GC staining. These data clearly show that the fibrosis-associated NO-GC-positive cells do not origin from the SMMHC lineage.

**Figure 6:**
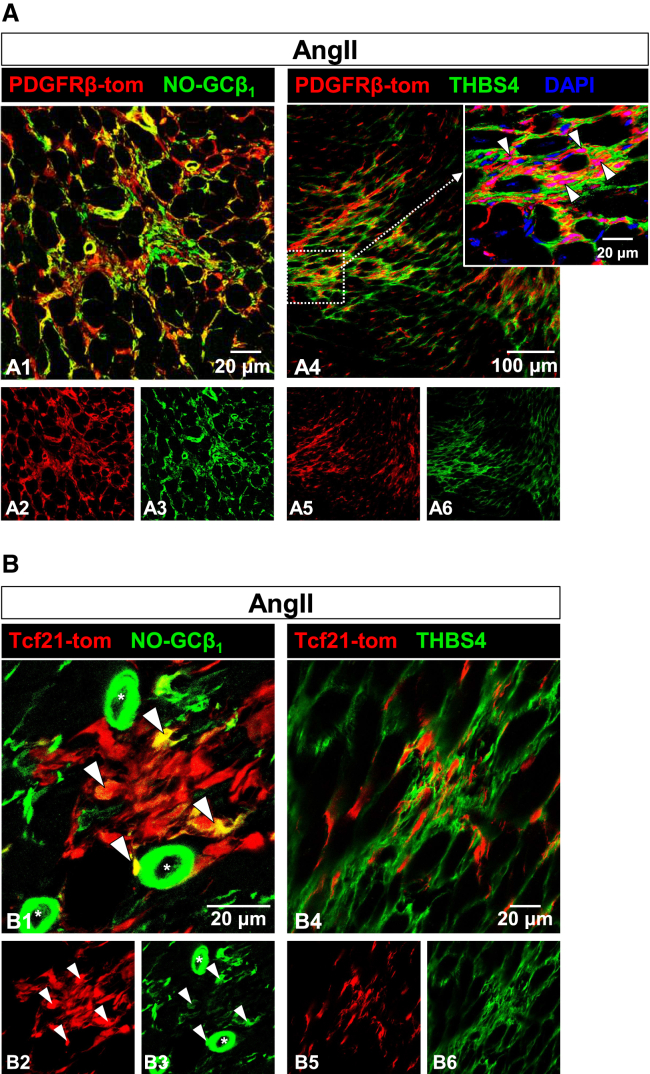
Lineage tracing of PDGFRβ-tomato and Tcf21-tomato cells in cardiac fibrosis Expression of tdTomato was tamoxifen-induced under control of PDGFRβ-CreER^T2^ (A) and Tcf21^mCrem^ (B). Subsequently, mice were treated with AngII for 2 weeks. (A) NO-GCβ_1_ (green) is found in most PDGFRβ-tdTomato-expressing cells both within and outside the fibrotic area. Cells within fibrotic area are surrounded by THBS4. DAPI (blue) is included in the enlargement of a4 to better show closeness of tdTomato expressing cells (purple nuclei) and THBS4. (B) NO-GCβ_1_ (green) is found in Tcf21-tdTomato-expressing cells (white arrowheads). Note that NO-GCβ_1_ immunosignals are weaker in these Tcf21-tdTomato-positive fibroblasts than in VSMC (marked by asterisks; negative for Tcf21-tdTomato) which is in accordance with scRNA data (see Figure 3C). Tcf21-tdTomato-positive fibroblasts are closely associated with THBS4 (b4). Single channels are shown in a2/3/5/6 and b2/3/5/6.

To corroborate the assumption that NO-GC-positive cells can originate from PDGFRβ- and Tcf21 lineages, we sought to show co-localization of the respective tdTomato reporter and NO-GC after AngII treatment. NO-GC was clearly detected in PDGFRβ-tdTomato-positive cells (Figure 6A). Expectedly, these cells were surrounded by THBS4 immunosignal (Figure 6A4; see also enlargement). NO-GC was also detected in Tcf21-tdTomato-expressing cells (Figure 6B). Here, NO-GC expression was fainter than in vascular SMC (compare green signals from cells marked with white arrowhead with those in vascular structures marked by asterisks). As seen with PDGFRβ-CreER^T2^, the Tcf21-tdTomato positive cells were surrounded by THBS4.

**Figure 7:**
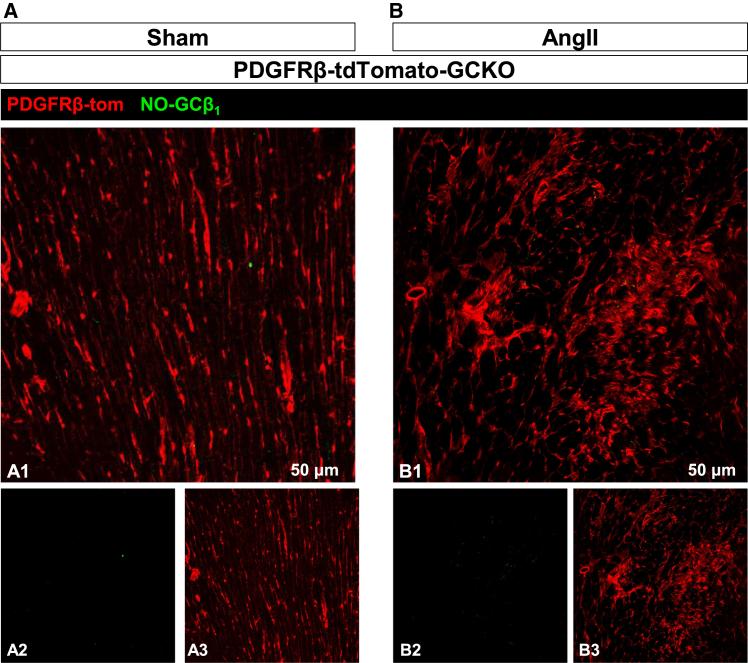
Absence of NO-GC immunosignals in PDGFRβ-CreER^T2^-mediated GCKO in sham- and AngII-treated heart PDGFRβ-CreER^T2^ was used to induce the tdTomato reporter and delete NO-GC at the same time (PDGFRβ-tdTomato-GCKO). Heart sections were stained for NO-GCβ_1_ (green), tdTomato is shown in red. The NO-GCβ_1_ immunosignal was absent in both healthy (A) and fibrotic heart. (B) Thus, NO-GC-expressing activated fibroblasts belong to both Tcf21 and PDGFRβ lineages. Images were acquired with a Leica SP8 confocal microscope. Single channels are shown in a2/3 and b2/3.

In a final approach, we used the PDGFRβ-CreER^T2^ to induce the tdTomato reporter and delete NO-GC at the same time (PDGFRβ-tdTomato-GCKO). PDGFRβ-CreER^T2^-mediated deletion of NO-GCβ_1_ in cardiac tissue was complete (Figure 7A). This finding corroborates the results in Figure 4A2–A5 which show that in the PDGFRβ/tdTomato reporter mouse, all cells labeled by the NO-GCβ_1_ antibody were tdTomato-positive. AngII treatment led to cardiac fibrosis in these mice which was devoid of NO-GC (Figure 7B). Therefore, the Tcf21-derived NO-GC-expressing fibroblasts must be part of the PDGFRβ lineage.

In conclusion, in the healthy myocardium NO-GC is predominantly expressed in pericytes and vascular smooth muscle cells, both of which are positive for PDGFRβ. Under fibrotic conditions, these cells do not contribute to the pool of NO-GC-positive activated fibroblasts. Rather, activated fibroblasts acquire NO-GC expression *de novo* during fibrosis. Comparison with scRNA-seq data indicate that these cells are the THBS4/NO-GC-expressing cells that arise upon AngII treatment. Our lineage tracing data allow us to conclude that these cells origin from PDGFRβ/Tcf21-positive lineage precursors.

## Discussion

This study confirms and builds upon previous reports of NO-GC expression in cardiac pericytes^10^ and VSMC.^24^ Notably, NO-GC colocalizes with PDGFRβ and αSMA (Figures 1B and 1D). The specificity of the antibody targeting the NO-GCβ_1_ subunit, identification of both NO-GC1 and NO-GC2 isoenzymes, was confirmed through its use in GCKO mice, as evidenced by the absence of binding (Figure S1). The identification of NO-GC-positive cells was further validated using an NO-GCα_1_-eGFP transgenic mouse model, in which the expression of enhanced green fluorescent protein (eGFP) is driven by the promoter activity of the α_1_ subunit of NO-GC. This allowed for the visualization of cells expressing the NO-GC1 isoform (Figure 1E). All eGFP-expressing cells were labeled by the NO-GCβ_1_ antibody, confirming the antibody’s sensitivity. Interestingly, some cells expressed the β_1_ subunit in the absence of NO-GCα_1_-eGFP, suggesting the presence of the NO-GC2 isoform. Sadly, there is no α_2_-specific antibody available to prove the subunit’s expression in these cells. Thus, the NO-GC1 isoform was found to be significantly more abundant in the heart than the NO-GC2 isoform. NO-GC expression in fibroblasts was undetectable by immunofluorescence in healthy myocardium, as judged from the absence of co-localization between NO-GC and PDGFRα (Figure 1C); in fact, scRNA-seq analysis corroborated very low expression levels in resident fibroblasts (Figure 3A). Collectively, these results indicate a robust expression of NO-GC in pericytes and VSMC, with only minimal expression in resident fibroblasts.

NO-GC expression in cardiomyocytes was undetectable in this study with our immunohistochemical methods (Figure 2), consistent with scRNA-seq data (Figure 3A). Unlike the previously reported colocalization of NO-GC1 with connexin 43 at the intercalated disc,^25^ we did not observe such staining. However, assuming that myosin heavy chain 6 (MYH6) is exclusively expressed in cardiomyocytes, the MYH6-GCKO mouse demonstrated functionally relevant NO-GC in these cells.^26^ Contrarily, NO-releasing compounds and NO-GC stimulators failed to increase cGMP levels in cardiomyocytes.^27^ These conflicting findings highlight the need for further investigation into NO-GC’s expression in cardiomyocytes.

Following AngII treatment, NO-GC expression was observed in fibrotic areas (Figure 3C). Additionally, NO-GC-positive cells in these areas were surrounded by the ECM protein THBS4 (Figure 3D). scRNA-seq analysis further confirmed GUCY1B1 RNA expression within the THBS4-positive fibroblast population, indicating NO-GC expression in activated fibroblasts (Figure 3A). THBS4, identified as a marker of activated fibroblasts,^16^ significantly increases following tissue injury.^15^^,^^28^ In humans, elevated THBS4 levels have been documented in the left ventricles of patients with heart failure^29^ and dilated cardiomyopathy,^30^ highlighting its role in cardiac fibrosis and disease progression. Similar results are shown in Figure S4 for the murine hypertrophic heart.

To compare our murine data to human hearts, we used publicly available single-nucleus RNA sequencing data comprising approximately 600,000 nuclei from left ventricular samples of 11 dilated cardiomyopathy hearts, 15 hypertrophic cardiomyopathy hearts, and 16 non-failing hearts.^31^ In non-failing human hearts, strong GUCY1B1 expression was observed in pericytes and VSMC (Figure S7), which corroborates our findings in mice. Additionally, GUCY1B1 expression was detected in resident fibroblasts, albeit at lower levels. In both dilated cardiomyopathy and hypertrophic cardiomyopathy, fibroblasts exhibited higher GUCY1B1 expression levels compared to those in non-failing hearts (Figures S7C and S7D). In fact, a distinct population of activated fibroblasts within these hearts expressed GUCY1B1, which is consistent with our findings in mice. Thus, the relevance of our data from the murine system is warranted and indicates translational importance in the human system.

### Origin of NO-GC-positive activated fibroblasts

After identifying NO-GC-positive activated fibroblasts in fibrotic regions, lineage tracing was conducted to investigate their origins. Two hypotheses were explored: the first proposes that NO-GC-expressing cells like pericytes migrate into fibrotic areas and differentiate into activated fibroblasts; the second suggests that precursor cells, such as resident fibroblasts, begin expressing NO-GC as they differentiate into activated fibroblasts. To examine these possibilities, lineage tracing was performed using the fluorescent protein tdTomato in combination with four CreER^T2^ mouse lines: PDGFRβ-, Tcf21-, SMMHC-, and NG2-CreER^T2^.

Our data revealed that PDGFRβ-tdTomato-positive cells accumulated in fibrotic areas (Figure 5A). While PDGFRβ is commonly used as a pericyte marker, its lack of specificity is well-known.^32^^,^^33^ In healthy myocardium, PDGFRβ-tdTomato also labeled SMC, (Figure 4A). Additionally, some of these PDGFRβ-tdTomato-positive cells expressed PDGFRα, a recognized fibroblast marker,^34^^,^^35^ as shown in Figure S6B. These findings, supported by scRNA-seq analysis (Figure 4A), indicate that PDGFRβ, while highly expressed in pericytes and SMCs, is also present in activated fibroblasts.

Tcf21-tdTomato-positive cells were also found in fibrotic areas (Figure 5B). Unlike PDGFRβ, Tcf21 primarily labels resident fibroblasts.^36^^,^^37^^,^^38^ It has been shown that following myocardial infarction, myofibroblasts originate from Tcf21-positive cells.^36^ Although Tcf21 is not entirely specific, it remains a reliable marker for resident fibroblasts in scRNA-seq and immunohistochemistry (Figures 4B and 6B). These results in combination with scRNA-seq trajectory analyses^16^ support the hypothesis that resident fibroblasts contribute significantly to the population of NO-GC-expressing activated fibroblasts in fibrotic regions.

Lineage tracing of SMMHC-tdTomato-positive cells demonstrated that SMC and most pericytes labeled by this reporter mouse (Figure 4D) do not migrate into fibrotic regions (Figures 5D and 5E). Thus, these cells do not appear to serve as precursors for activated fibroblasts. Similarly, NG2-tdTomato-positive cells, another marker for pericytes,^32^ did not migrate into fibrotic areas (Figure 5C), further suggesting that pericytes are not the primary source of activated fibroblasts. However, a small subpopulation of pericytes, characterized by PDGFRβ-tdTomato expression but lacking SMMHC- or NG2-tdTomato labeling, might still contribute to the formation of activated fibroblasts. A study on myocardial infarction using NG2 as a pericyte marker and PDGFRα as a fibroblast marker suggests that a subset of pericytes transiently expressed fibroblast markers, contributing to a minor fraction of fibroblasts.^39^ Conversely, research using the Tbx18-CreER^T2^ mouse and TAC model indicate that pericytes maintain their identity, with evidence suggesting that their apparent plasticity and potential role as mesenchymal stem cells *in vitro* may be due to artificial cell manipulation rather than reflecting their true role *in vivo*.^40^

Taken together, the collective immunofluorescence/scRNA-seq findings of this study, supported by existing literature,^15^ strongly suggest that resident fibroblasts are the primary origin of NO-GC-positive activated fibroblasts in fibrotic regions. The role of NO-GC in these cells has to be elucidated still, yet being a target for therapeutically used NO-GC stimulators like vericiguat to modulate tension within the fibrotic scar appears conceivable.

### Limitations of the study

Limitations of our study include the use of different Cre strains which may not be fully specific for the cells indicated, e.g., the SMMHC-Cre mouse was originally described as ‘SMC-specific’ but turned out later to also target pericytes. We are also aware that the AngII treatment induces a variety of symptoms including cardiac hypertrophy and fibrosis. Other models such as transverse aortic constriction or treatment with sympathomimetics may induce similar overall symptoms but may share different mechanisms which may lead to a qualitatively different remodeling.

## Resource availability

### Lead contact

Further information and requests for resources and reagents should be directed to and will be fulfilled by the lead contact, Dr. Andreas Friebe (andreas.friebe@uni-wuerzburg.de).

### Materials availability

Mice used in this study are available from lead contact under a material transfer agreement with the University of Würzburg.

### Data and code availability

• Data: All data reported in this paper will be shared by the lead contact upon request.
• Code: This paper does not report any code.
• Other items: Any additional information required to reanalyze the data reported in this paper is available from the lead contact upon request.

## Acknowledgments

The excellent technical help of Bianca Moran and Maria Gallant is gratefully acknowledged. The work was supported by 10.13039/501100001659Deutsche Forschungsgemeinschaft to AF (FR 1725/10-1) and by the 10.13039/501100001030National Heart Foundation of Australia to A.R.P. (Future Leader Fellowship Level 3, 107335).

## Author contributions

L.K. designed and performed experiments, analyzed the data, produced figures and wrote the manuscript. A.G. and D.G. performed experiments. A.R.P. provided scRNA-seq data and MDT provided materials. A.F. designed and conceptualized experiments, analyzed data, produced figures, wrote the manuscript, and acquired funding. All authors read the manuscript and provided critical comments.

## Declaration of interests

The authors declare no competing interests.

## STAR★Methods

### Key resources table

**Table:** 

REAGENT or RESOURCE	SOURCE	IDENTIFIER
**Antibodies**		

Anti-goat αSMA	Novus Biologicals	Cat#NB300-978
Anti-rat CD31	BD Pharmingen	Cat#550274
Anti-goat desmin	Antibodies-online	Cat#ABIN334386
Anti-rabbit NO-GCβ_1_	Friebe laboratory, own production	N/A
Anti-goat PDGFRα	R&D Systems	Cat#AF1062
Anti-goat PDGFRβ	R&D Systems	Cat#AF1042
Anti-goat THBS4	R&D Systems	Cat#MAB7860
donkey anti-rabbit IgG Alexa Fluor™ 488	Invitrogen	Cat#A21206
donkey anti-rat IgG Alexa Fluor™ 488	Invitrogen	Cat#A21208
donkey anti-rabbit IgG Alexa Fluor™ 555	Invitrogen	Cat#A31572
goat anti-rabbit IgG Alexa Fluor™ 633	Invitrogen	Cat#A21070
donkey anti-goat IgG Alexa Fluor™ 647	Invitrogen	Cat#A21447
chicken anti-rat IgG Alexa Fluor™ 647	Invitrogen	Cat#A21472

**Chemicals, peptides, and recombinant proteins**		

Tamoxifen	Sigma-Aldrich	Cat#T5648
Miglyol 812	Caesar & Loretz	Cat#3274
Angiotensin II human	Sigma-Aldrich	Cat#A9525
Isofluran CP®	CP-Pharma	N/A
Rimadyl® (Carprofen)	Zoetis Deutschland	N/A
Lidocaine hydrochloride 2%	Bela-pharm	N/A
Triton-X-100	Sigma-Aldrich	Cat#T8787
normal donkey serum	Biozol	Cat#LIN-END9000-100
DAPI	Applichem	Cat#A1001
Mowiol	Carl Roth	Cat#0713

**Experimental models: Organisms/strains**		

Floxed NO-GC (NO-GCβ_1_^flox/+^)	Friebe lab	N/A
PDGFRβ-CreER^T2^	Chen et al.^41^	N/A
TCF21^mCrem^	Acharya et al.^42^	N/A
SMMHC-CreER^T2^	Wirth et al.^43^	N/A
NG2-CreER^T2^	Zhu et al.^44^	N/A
PDGFRβ-control (PDGFRβ^+/-^;tdTomato^+/-^;NO-GCβ_1_^flox/+^)		N/A
PDGFRβ-GCKO (PDGFRβ^+/-^;tdTomato^+/-^;NO-GCβ_1_^flox/-^)		N/A
NO-GCα_1_-eGFP mouse (B6;FVB-Tg(Gucy1a3-EGFP)HM112Gsat/Mmucd)	MMRRC, UC Davis	stock Number 030080-UCD

**Software and algorithms**		

Leica Application Suite X Office Vers. 1.4.5	Leica Microsystems	N/A
ImageJ2 Vers. 2.14.0	Wayne Rasband	N/A

**Other**		

Leica cryostat CM3050S	Leica Microsystems	N/A
Leica confocal microscope (Leica TCS SP8)	Leica Microsystems	N/A
osmotic minipumps Alzet Model 1002	Charles River	Cat#0004317
ImmEdge pen	Vector Laboratories	VEC-H-4000

### Experimental model and study participant details

#### Mice

All animal experiments in this study conform to the Animal Research: Reporting of *In Vivo* Experiments (ARRIVE) guidelines (http://www.nc3rs.org.uk/arrive-guidelines). All experimental procedures received approval from the local animal care committee (Bezirksregierung Unterfranken; application numbers: 55.2.2-2532-2-1108, -233, -1365, -1414 and -1537), in accordance with § 8, section 1 of the German Animal Welfare Act and the European Directive 2010/63/EU. Mice were housed in a secure, access-controlled rodent facility with strictly regulated environmental conditions. Animals were kept in type II macrolon cages (335 cm^2^, accommodating up to three adults) or type IIL macrolon cages (540 cm^2^, accommodating up to five adults), with coarse wooden bedding. A standard rodent diet (Altromin, Lage, Germany) and water were provided *ad libitum*. For global guanylyl cyclase knockout (GCKO) mice, a specialized low-fiber diet supplemented with esomeprazole and bicarbonate was administered to mitigate reduced gastrointestinal motility and potential ulceration; this regimen was also applied to their control group siblings. Environmental enrichment was ensured by providing cellulose nesting material and small shelters in all cages. The facility maintained a stable environment, with a 12-hour light/dark cycle, ambient temperatures between 20°C and 24°C, and relative humidity between 45% and 65%. Routine husbandry included weekly cage cleaning and water bottle replacement. A total of 78 animals of both sexes was used.

The NO-GCα_1_-eGFP mouse (B6;FVB-Tg(Gucy1a3-EGFP)HM112Gsat/Mmucd) was obtained from MMRRC, UC Davis, stock Number 030080-UCD.

In the PDGFRβ-tdTomato-GCKO, the PDGFRβ-CreER^T2^ induces deletion of NO-GC and, in parallel, expression of the tomato reporter. This line was generated by breeding PDGFRβ-tdTomato mice (PDGFRβ^+/-^;tdTomato^+/-^) heterozygous for NO-GCβ_1_ (NO-GCβ_1_^+/-^) with mice carrying homozygously floxed NO-GCβ_1_ (NO-GCβ_1_^flox/flox^). Thus, mice used as PDGFRβ-tdTomato-GCKO were PDGFRβ^+/-^;tdTomato^+/-^;NO-GCβ_1_^flox/-^. Tamoxifen injection then induced deletion of the floxed NO-GCβ_1_ exon and induction of tdTomato.

#### Generation of tamoxifen-inducible promoter-specific tdTomato reporter mice

The mice utilized in this study were on C57BL/6 or mixed C57BL/6/129SV genetic background. Breeding pairs consisted of two female mice (aged 8-45 weeks) and one male mouse (aged 7-52 weeks). Offspring were weaned at 18-21 days of age and subsequently genotyped. A Cre recombinase tdTomato reporter system was employed for lineage tracing (Ai14; JAX #007914). This reporter line was crossed with four distinct Cre-expressing mouse lines: PDGFRβ-CreER^T2^,^41^ TCF21^mCrem^,^42^ SMMHC-CreER^T2^,^43^ and NG2-CreER^T2^.^44^ Notably, in the SMMHC-CreER^T2^ line, the CreER^T2^ gene was located on the Y chromosome, resulting in exclusive expression in male offspring. Consequently, only male mice were included in this study.

### Method details

#### Tamoxifen administration

Tamoxifen (Sigma, Taufkirchen, Germany) was prepared at a concentration of 20 mg/ml in Miglyol 812 (capric acid triglyceride), and each mouse received an intraperitoneal injection of 1 mg tamoxifen in a 50 μl volume on five consecutive days at six weeks of age.

#### Osmotic minipumps

At 13 weeks of age, osmotic minipumps (Alzet Model 1002; Charles River, Sulzfeld, Germany) delivering 2 mg/kg/day of AngII or 0.9% NaCl (control) were implanted subcutaneously. These pumps administered AngII/NaCl continuously for 14 days, after which hearts were harvested for analysis. All minipump handling and filling procedures were performed under sterile conditions in a class II microbiological safety cabinet.

Anesthesia was induced using 4% isoflurane in oxygen at 1 l/min within an induction chamber and maintained at 2% via a nasal cone. Analgesia was provided through an intraperitoneal injection of 5 mg/kg carprofen, and eye ointment was applied to prevent corneal drying. For local analgesia, 4 mg/kg lidocaine was administered subcutaneously between the scapulae. The surgical site on the upper back was shaved and disinfected. After confirming sufficient anesthesia via the interdigital reflex, a 5 mm transverse incision was made between the scapulae. A subcutaneous pocket was then created, and the minipump, oriented with the flow moderator tailward, was inserted. The incision was closed with single button sutures.

#### Immunofluorescence

Following cervical dislocation and thoracic cavity exposure, the descending thoracic aorta was clamped, and 1 ml of 2% paraformaldehyde (PFA) was injected retrogradely above the clamp. The heart was subsequently excised, with the atria removed and excess liquid carefully drained. The heart was then bisected longitudinally and fixed in 2% PFA for 1 hour, rinsed in phosphate-buffered saline (PBS) for 30 minutes, and incubated overnight in 20% sucrose at 4°C. For thermal fixation, 100 ml of isopentane was cooled in liquid nitrogen. The heart, dried briefly on a paper towel, was immersed in the cooled isopentane for 20 seconds. The sample was then placed on dry ice for 15 minutes to allow isopentane evaporation, followed by storage at -80°C.

Tissue samples were sectioned at a thickness of 20 μm using a cryostat and immediately transferred onto slides. The sections were then outlined with an ImmEdge pen before proceeding with immunohistochemistry. All solutions were sterile filtered before use. Sections were fixed in 4% PFA for 10 minutes, then washed three times with PBS for 10 minutes each. Permeabilization was performed using 0.5% Triton-X for 20 minutes, followed by two additional PBS washes. To reduce non-specific binding, sections were incubated with 10% normal donkey serum (NDS) before being incubated with primary antibodies (see the table below) overnight at 4 °C. The following day, sections were washed three times with PBS and incubated with secondary antibodies (1:500 dilution; see the table below) at room temperature, shielded from light. Nuclear staining was performed using DAPI (1:1000 dilution in PBS; Applichem, Heidelberg, Germany) for 7 minutes, followed by three PBS washes. Slides were then mounted with Mowiol and imaged using a Leica confocal microscope (Leica TCS SP8). Images with thicknesses ranging from 1-10 μm were generated using Z-stacks.

**Table:** Antibodies

Antibody	Organism	Dilution	Company	cat no
**Primary antibodies**				

αSMA	goat	1:200	Novus Biologicals, Centennial, CO, USA	NB300-978
CD31	rat	1:200	BD Pharmingen, San Diego, CA, USA	550274
desmin	goat	1:200	Antibodies-online, Aachen, Germany	ABIN334386
NO-GCβ_1_	rabbit	1:500	Friebe laboratory, own production	–
PDGFRα	goat	1:200	R&D Systems, Minneapolis, MN, USA	AF1062
PDGFRβ	goat	1:200	R&D Systems, Minneapolis, MN, USA	AF1042
THBS4	rat	1:200	R&D Systems, Minneapolis, MN, USA	MAB7860

**Secondary antibodies**				

Alexa Fluor™ 488	donkey anti-rabbit IgG	1:500	Invitrogen, Waltham, MA, USA	A21206
Alexa Fluor™ 488	donkey anti-rat IgG	1:500		A21208
Alexa Fluor™ 555	donkey anti-rabbit IgG	1:500		A31572
Alexa Fluor™ 633	goat anti-rabbit IgG	1:500		A21070
Alexa Fluor™ 647	donkey anti-goat IgG	1:500		A21447
Alexa Fluor™ 647	chicken anti-rat IgG	1:500		A21472

#### scRNA-seq analysis

Single-cell RNA sequencing (scRNA-seq) data used in this study were obtained from,^16^ with full methodological details outlined in the original publication. The data are publicly accessible, including through the Gene Expression Atlas at https://www.ebi.ac.uk/gxa/sc/experiments/E-MTAB-8810/results/cell-plots.
