# Supplementary material for: Analysis of cellular NO-GC expression in the murine heart and lineage determination in angiotensin II-induced fibrosis
Source: iScience. 2024 Dec 16;28(1):111615. doi: 10.1016/j.isci.2024.111615 (PMC11742323; doi:10.1016/j.isci.2024.111615)
Supplement: Document S1. Figures S1–S7 [file mmc1.pdf]

iScience, Volume *28*

Supplemental information

Analysis of cellular NO-GC expression in the murine heart and lineage determination in angiotensin II-induced fibrosis

Lennart Kreutz, Annika Gaab, Malathi Dona, Alexander R. Pinto, Michelle D. Tallquist, Dieter Groneberg, and Andreas Friebe

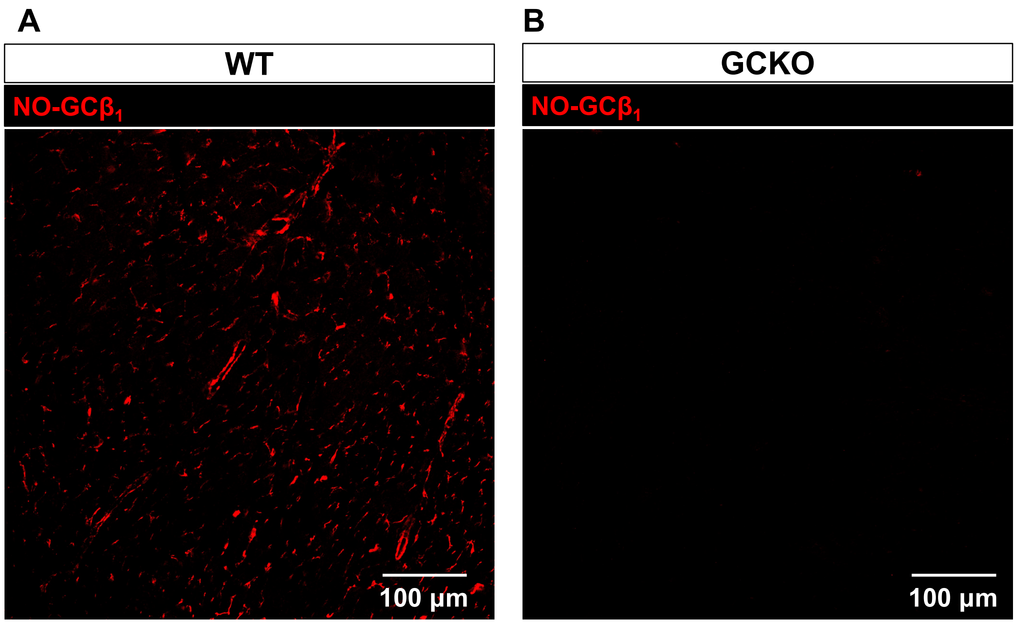

Figure S1: Absence of NO-GC immunosignals in GCKO reveals specificity of NO-GCβ1 antibody

Panel **A** shows the NO-GCβ_1 antibody signal in WT mice in red. In global NO-GC knockout (GCKO) mice the immunosignal was absent (**B**), confirming the antibody's specificity. Images were acquired with a Leica SP8 confocal microscope with a 20x objective. Immunohistochemistry was conducted using identical chemicals, and images were captured with identical settings for laser intensity, gain, and emission filter, all on the same day.

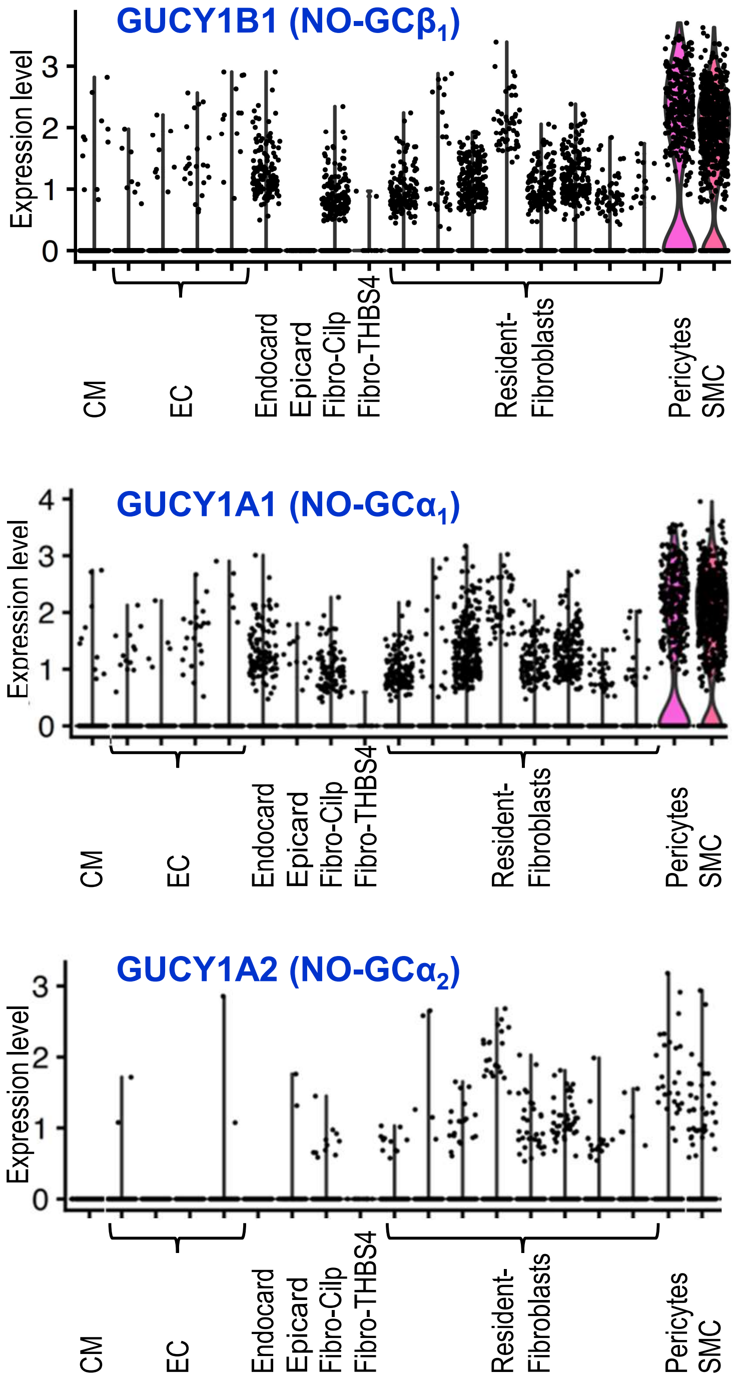

Figure S2: Cardiac RNA expression of NO-GC subunits

Violin plots illustrating scRNA expression of the three known NO-GC subunits (GUCY1B1: β_1 subunit; GUCY1A1: α_1 subunit; GUCY1A2: α_2 subunit). A strong expression of the $\alpha_1\beta_1$ isoform is evident in pericytes and smooth muscle cells whereas α_2 subunit expression is rather low in all cells. Very minor NO-GC presence is observed in the endocardium, epicardium and endothelium whereas low expression can be found in resident fibroblasts. Cardiomyocytes (CMs), endothelial cells (EC), endocardium (Endocard), epicardium (Epicard), fibroblasts (Fibro), Pericytes, smooth muscle cells (SMC). scRNAseq data from McLellan et al., 2020; https://www.ebi.ac.uk/gxa/sc/experiments/E-MTAB-8810/results/tsne.

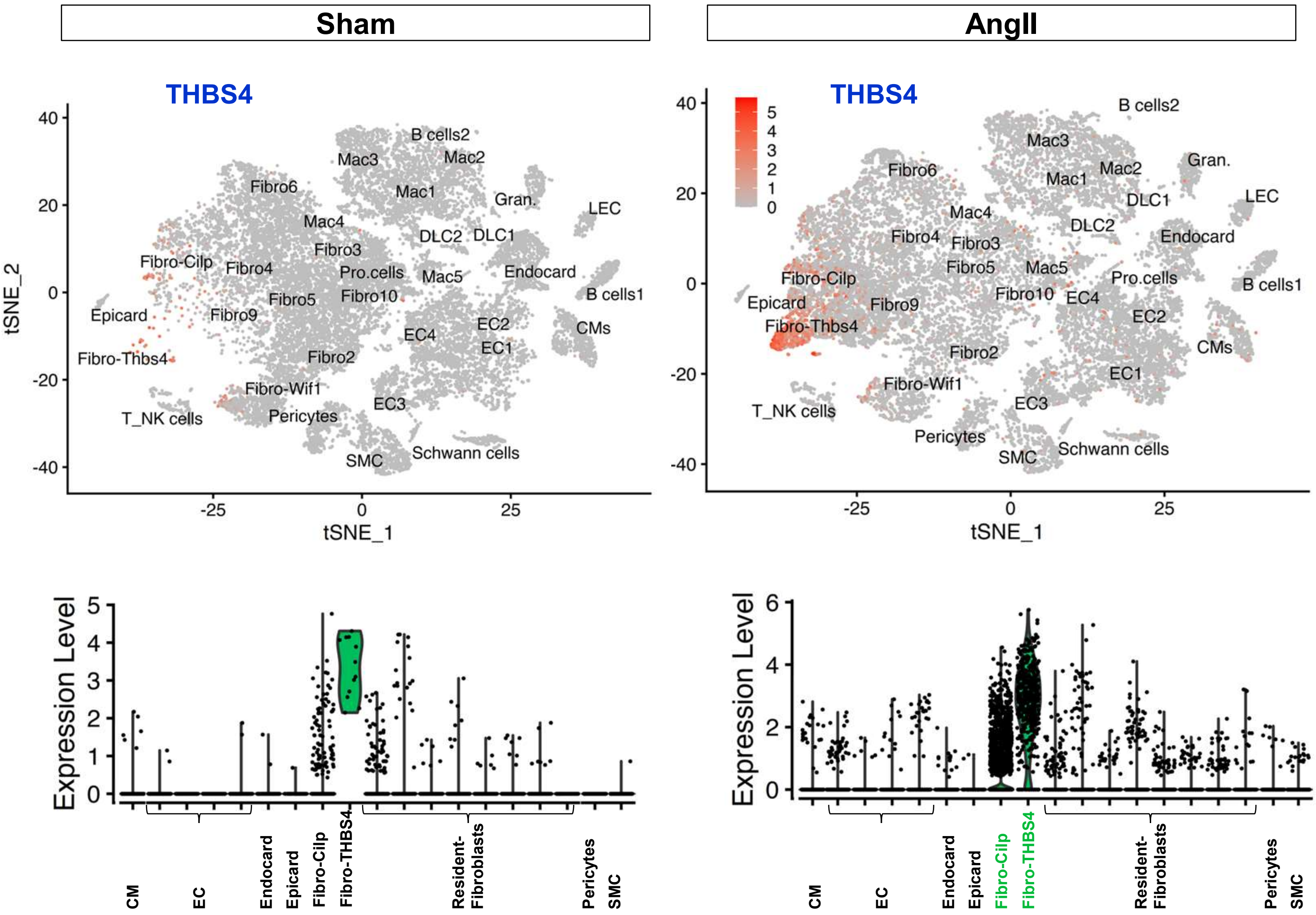

Figure S3: Cardiac RNA expression of THBS4

Violin plots illustrating scRNA expression of THBS4. Under non-fibrotic conditions, relevant THBS4 expression is evident in very few fibroblasts. After AngII treatment, newly formed THBS4-fibroblasts in conjunction with Cilp-fibroblasts show strong THBS4 expression. The tSNE diagram indicates that both fibroblast subtypes are strongly increased in number after AngII treatment. Cardiomyocytes (CMs), endothelial cells (EC), endocardium (Endocard), epicardium (Epicard), fibroblasts (Fibro), Pericytes, smooth muscle cells (SMC). scRNAseq data from McLellan et al., 2020; https://www.ebi.ac.uk/gxa/sc/experiments/E-MTAB-8810/results/tsne.

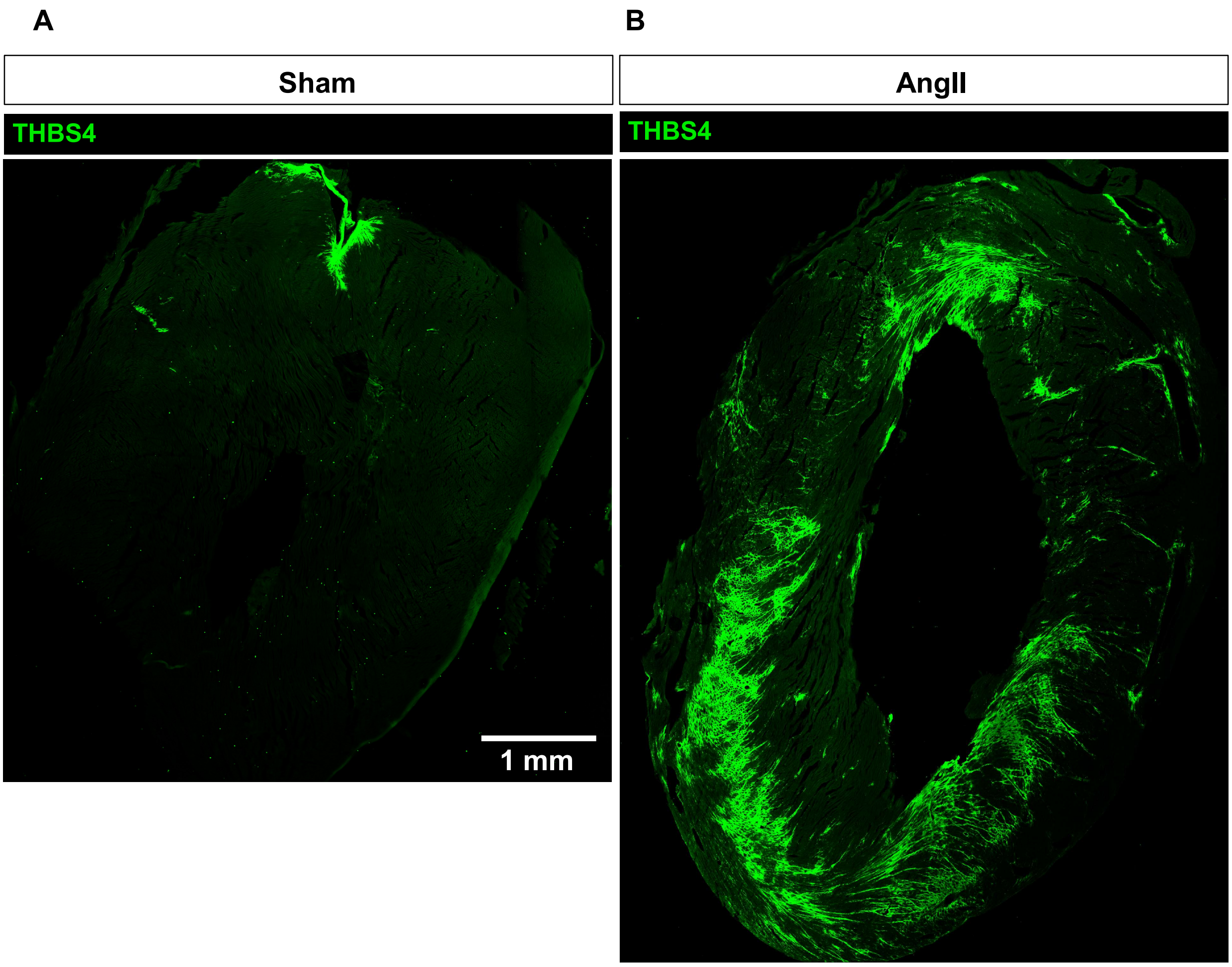

Figure S4: THBS4 labeling in whole hearts of sham- and AngII-treated mice

THBS4 (green) is shown in longitudinal heart sections. In sham-treated mice (**A**), THBS4 marks the annulus of the aortic valve, with no labeling detected within the cardiac tissue. Hearts from AngII-treated mice (**B**) exhibit extensive THBS4 labeling of fibrotic areas, demonstrating its specificity and the extensive fibrosis induced by AngII treatment. Notably, cardiac hypertrophy is evident in the AngII-treated heart. Images were acquired with a Leica SP8 confocal microscope with a 10x objective, employing an XY-stack imaging technique to comprehensively visualize the entire heart section. To visualize the overall structure of the heart, the filter was opened wide to allow detection of autofluorescence.

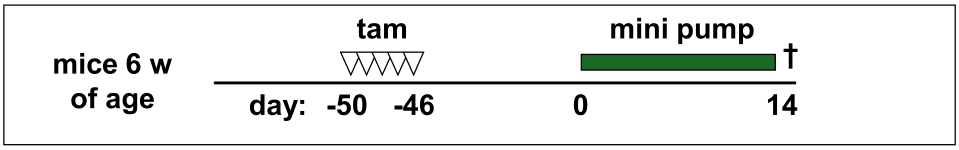

Figure S5: Timeline of tamoxifen injection and minipump application

Mice aged 6 weeks were injected with tamoxifen on 5 consecutive days to induce the expression of tdTomato under control of the respective promoter. 45 days later mini-pumps containing either saline or AngII (2 mg/kg/d) were implanted. After 14 days, mice were sacrificed, and hearts were collected.

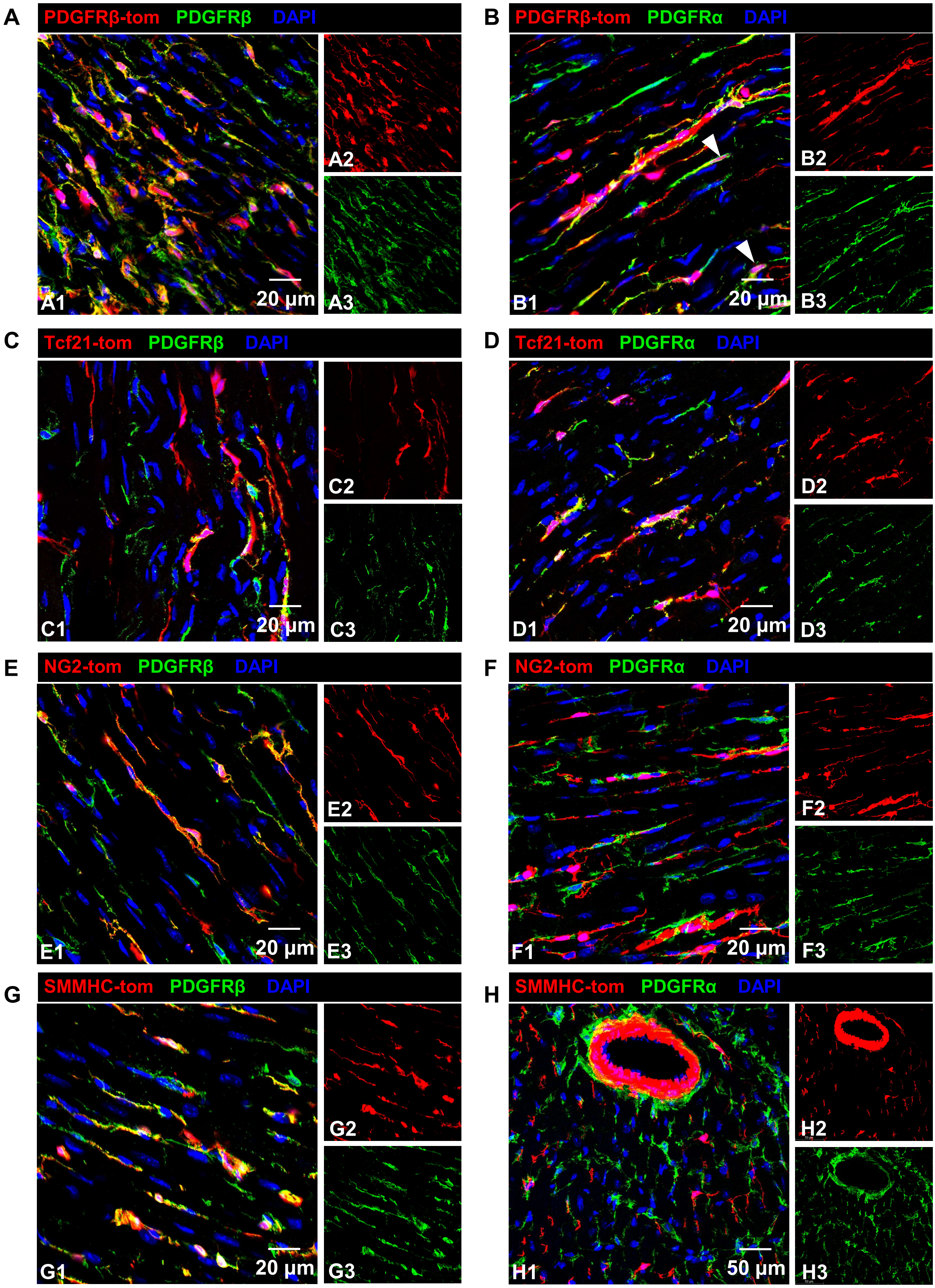

Figure S6: Co-localization of PDGFRβ and PDGFRβ with Cre line-dependent tdTomato staining

Heart sections from PDGFRβ-tdTomato (A and B), Tcf21-tdTomato (C and D), NG2-tdTomato (E and F), and SMMHC-tdTomato (G and H) mice were stained with antibodies against PDGFRβ and PDGFRα.

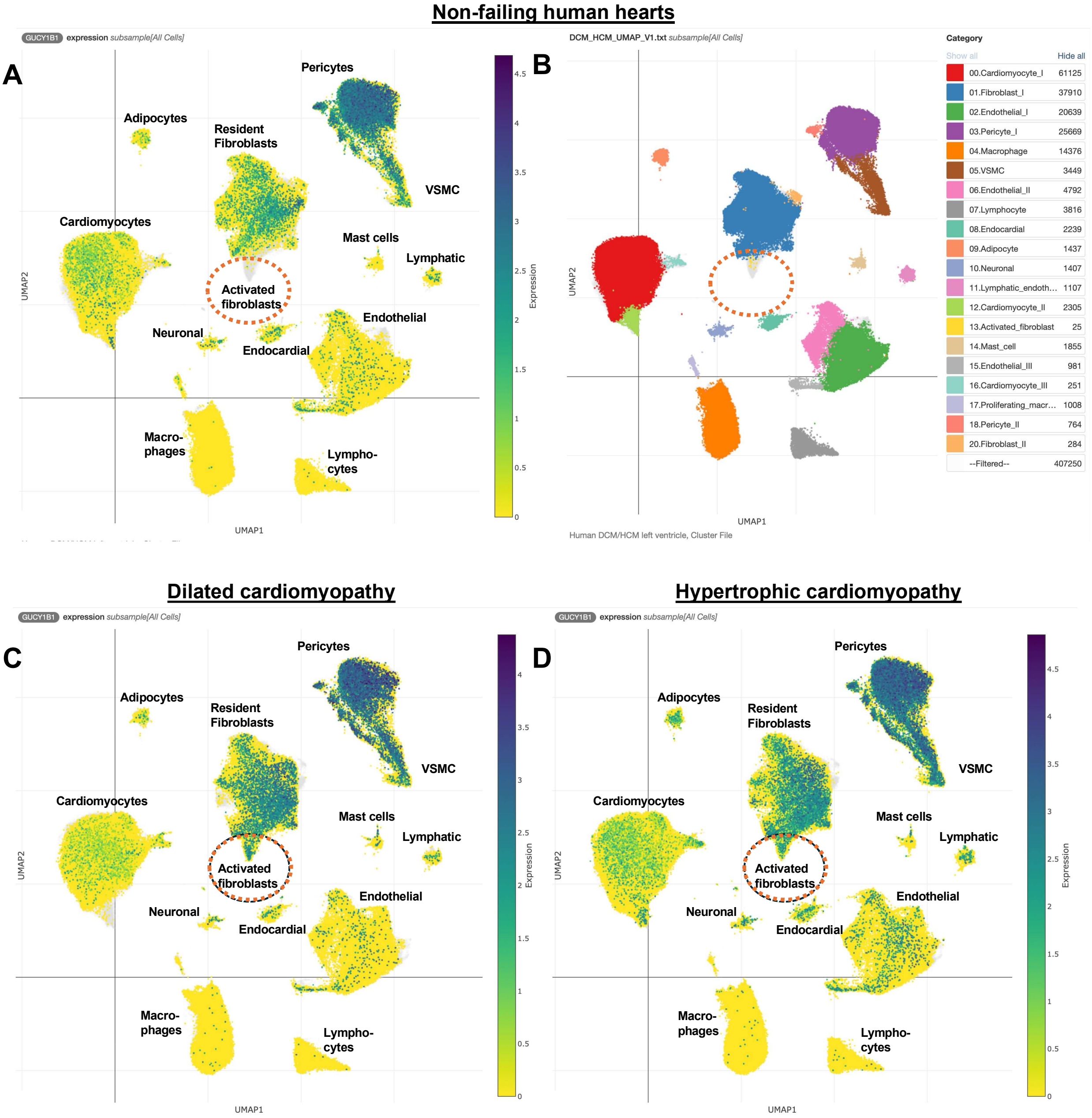

Figure S7: GUCY1B1 single-nucleus RNA expression of non-failing human hearts and human hearts with dilated and hypertrophic cardiomyopathy

(**A**) Single-nucleus RNA sequencing data illustrating the expression of the ß1 subunit of NO-GC (GUCY1B1) in human heart tissue. (**B**) Legend identifying the various cell populations. GUCY1B1 expression is notably strong in pericytes and vascular smooth muscle cells (VSMCs), and is also present, though at lower levels, in resident fibroblasts.

Single-nucleus RNA from human hearts with dilated cardiomyopathy (**C**) and hypertrophic cardiomyopathy (**D**) reveals strong GUCY1B1 expression in pericytes and vascular smooth muscle cells, as well as in both resident and activated fibroblasts (highlighted by dotted circle). Data from Chaffin et al., 2022; https://singlecell.broadinstitute.org/single_cell/study/SCP1303/single-nuclei-profiling-of-human-dilated-and-hypertrophic-cardiomyopathy (modified).
